# Single-step purification of intrinsic protein complexes in *Saccharomyces cerevisiae* using regenerable calmodulin resin

**DOI:** 10.1016/j.mex.2018.06.005

**Published:** 2018-06-12

**Authors:** Urvi Bhojoo, Kyle K. Biggar

**Affiliations:** a Institute of Biochemistry, Carleton University, 1125 Colonel By Drive, Ottawa Ontario, K1N 5B6, Canada; b Department of Biology, Carleton University, 1125 Colonel By Drive, Ottawa Ontario, K1N 5B6, Canada

**Keywords:** TAP-tag, Matrix regeneration, *Saccharomyces cerevisiae*, COMPASS complex, Swd3-TAP, Set1

## Abstract

Characterization of protein- protein interactions is a vital aspect of molecular biology as multi-protein complexes are the functional units of cellular processes and defining their interactions provides valuable insights into their function based on a “guilt by association” concept. To identify the components in complexes, purification of the latter near homogeneity is required. The tandem affinity purification (i.e., TAP) method, coupled with mass spectrometry have been extensively used to define native protein complexes and transient protein-protein interactions under near physiological conditions (i.e., conditions approximate of the internal milieu) in *Saccharomyces cerevisiae.* Generally, TAP consists of two-stage protein enrichment using dual affinity tags, a calmodulin-binding peptide and a *Staphylococcus aureus* protein-A, separated by a tobacco etch virus protease site, which are fused to either the C- or N-terminal of the target protein. TAP-tagging has proved to be a powerful method for studying functional relationship between proteins and generating large-scale protein networks. The method described in this paper provides an inexpensive single-step purification alternative to the traditional two step affinity purification of TAP-tagged proteins using only the calmodulin-binding peptide affinity tag. Moreover, a novel protocol for the regeneration of the calmodulin-agarose resin is outlined and validated. This basic approach allows fast and cost-effective purification of proteins and their interacting partners from *Saccharomyces cerevisiae*.

## Graphical abstract

**Figure:**
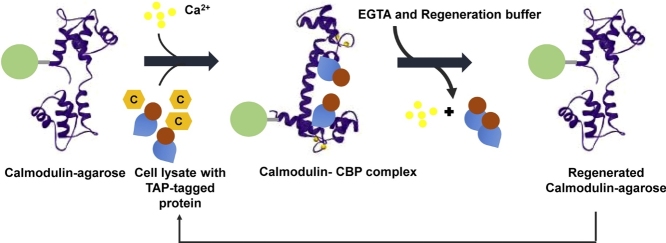

Specification table

**Table:** 

Subject area	Biochemistry, Genetic and Molecular Biology
More specific subject area	Proteomics, protein purification
Protocol name	Regenerable single-step purification of protein complexes
Name and reference of original method	Rigaut, G. ; Shevchenko, A. ; Rutz, B. ; Wilm, M. ; Mann, M. ; Séraphin, B. A Generic Protein Purification Method for Protein Complex Characterization and Proteome Exploration. *Nat. Biotechnol.***1999**, *17* (10), 7–9.
Resource availability	• calmodulin-agarose beads (Sigma-Aldrich) • snake Skin dialysis tubing (Thermo Scientific)

## Value of the protocol

• Simple method to identify *in vivo* protein complexes.
• Fast and cost-effective single-step purification of TAP-tagged protein complexes that are expressed under physiological conditions.
• Protocol for the regeneration of calmodulin resin for TAP.

## Protocol background

Proteins have crucial roles in biological processes. It is well-established that these functional units execute their jobs by either interacting with themselves or through multiple interactions with other proteins or molecules including DNA and lipids and functioning as a complex. Moreover, protein interactions also dictate the mechanisms which determine the phenotype of the organism [1]. It has been demonstrated in several instances that pathological conditions such cancer and neurodegenerative disease often result from protein dysregulation and dysfunction [[1], [2], [3]]. Therefore, it is critical to define and understand protein functions and interactions. Proteome analysis necessitates fast and efficient purification techniques [4]. To this end, affinity tags involving the fusion of a peptide or a small protein to the bait protein have been extensively used for protein purification.

**Fig. 1:**
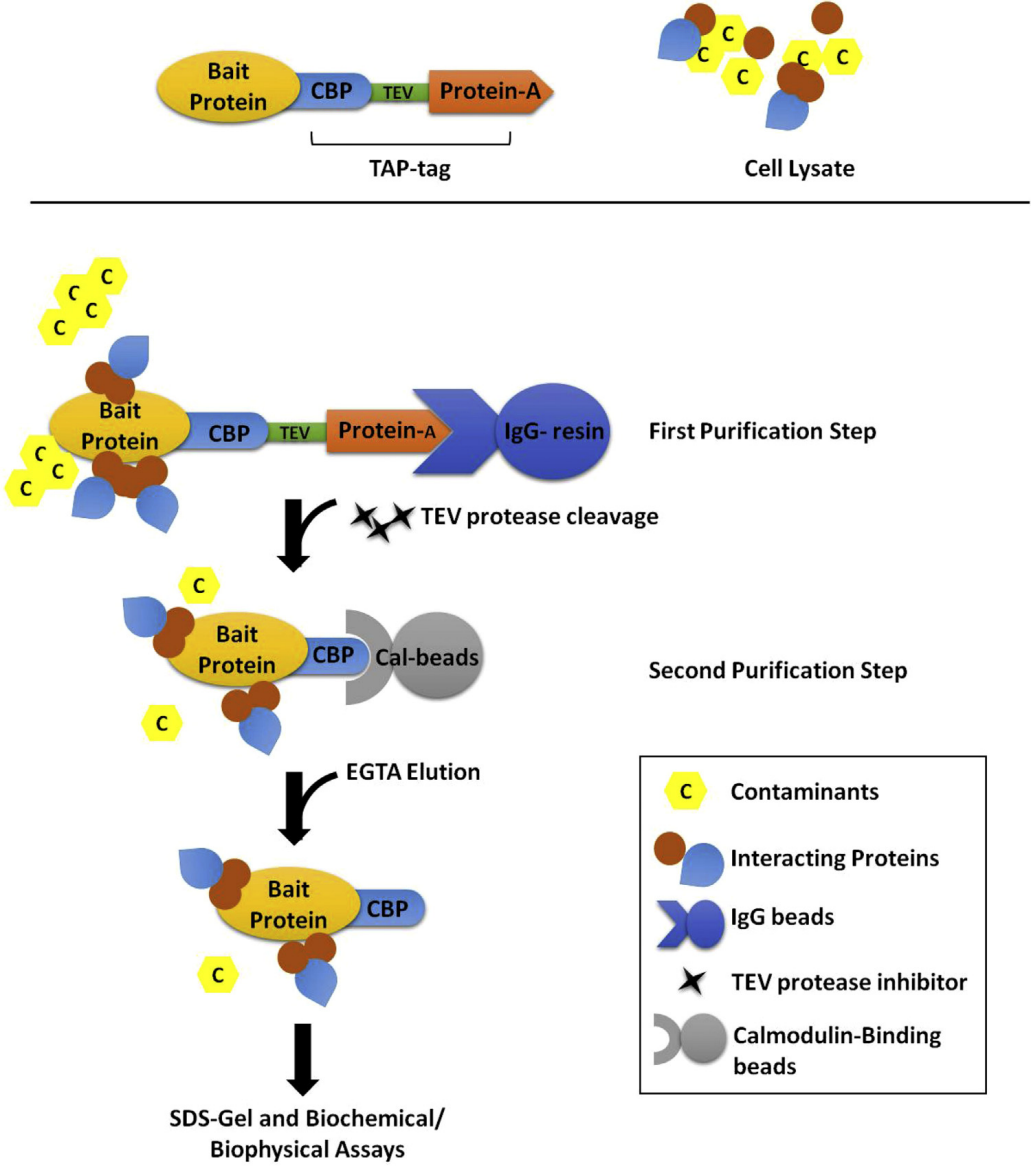
Schematic representation of the steps involved in the two-step purification of TAP-tagged protein. The affinity tag is composed of a Calmodulin-binding protein (CBP) followed by a tobacco etch virus (TEV) restriction and *Staphylococcus aureus* Protein-A. In the first round of protein enrichment, Protein-A of the affinity tag interacts with the immunoglobulin G (IgG) resin, separating the bait protein and its interacting partners from contaminating proteins. TEV restriction enzymes elute the bait protein from the IgG resin. In the second step, CBP tag further reduces the presence of co-contaminants by binding to the calmodulin resin in the presence of calcium. ETGA elution releases the bait protein and the co-purified proteins which are subsequently run on an SDS-PAGE gel and analyzed by mass spectroscopy or used in biochemical assays.

In *Saccharomyces cerevisiae,* TAP-tagging has proved to be efficacious in separating native protein complexes and transient protein interactions, at near physiological conditions [[5], [6], [7]]. The TAP-tag consist of two components, a calmodulin-binding protein (CBP) and an IgG binding domain of *Staphylococcus aureus* Protein-A, which are separated by a TEV cleavage site (Fig. 1). In the first purification stage, protein-A interaction with IgG resin retains the bait protein while contaminants elute from the column. The release of the bait protein from the IgG resin requires denaturing conditions at low pH or proteolytic cleavage under native conditions. In the second stage, the CBP tag separates the bait protein from any remaining cell lysate contaminants [4,8]. The interaction of CBP to calmodulin beads is calcium-dependent. Consequently, the elution of the target protein requires only a calcium chelator such as EGTA [9]. The TAP-tagging method has been widely used for generating protein interactomes through mass spectrometry [[10], [11], [12]].

However, the high cost of the calmodulin resin as compared to other non-protein resins can discourage the utilization of TAP-tags for biochemical and biophysical assays which require considerable amounts of purified proteins. The method described below provides an optimized protocol for one-step purification of TAP-tagged protein from *S. cerevisiae* under non-denaturing conditions using CBP part of the affinity tag. Moreover, we describe a validated procedure for the regeneration of the calmodulin resin.

## Method details

### Step 1: culturing and harvesting *S. cerevisiae*

#### Materials

• Sterile YPD Media (10 g yeast extract, 20 g peptone and 20 g D-glucose in 1 L distilled water).
• IPBL buffer (20 mM HEPES KOH (pH 7.4), 150 mM KOAc, 2 mM Mg(Ac)_2_, 1 mM EGTA and 10% glycerol).
• Liquid nitrogen.
• Sterile 15 mL, 100 mL and 2 L flasks.
• Sterile pipette tips.

#### Procedure

1 Using sterile techniques, inoculate TAP-tagged *S. cerevisiae* strain (from glycerol stock or cells grown on YPD-agar plate) in 5 mL of sterile YPD liquid medium in a 15 mL culture tube. Grow the yeast culture overnight at 30 °C in a shaking incubator at 250 rpm.
2 Inoculate 5 mL of the overnight culture into 40 mL YPD media in 100 mL sterile flask and allow cells to grow overnight at 30 °C and 250 rpm.
3 Add 10–20 mL of the overnight culture to 300 mL YPD media and grow the culture at 30 °C with shaking at 250 rpm until an OD_600_ of 1.0–1.2.
4 Transfer the 300 mL into clean centrifugation bottles and centrifuge culture for 5 min. at 5000 rpm.
5 Discard the supernatant taking care not to disturb the pellet. Add 10 mL of cold distilled water (4 °C) to the bottle to re-suspend the pellet by swirling or pipetting.
6 Transfer the cell suspension into a clean 50 mL conical tube and centrifuge the latter for 5 min. at 3000 rpm at 4 °C.
7 Discard the supernatant and wash pellet 2 more times- first with 10 mL cold distilled and then with 10 mL IPBL buffer, without protease inhibitor.
8 Briefly invert the 50 mL-tube on paper towel to remove any remaining liquid, careful not to lose the pellet.
9 Using liquid nitrogen, flash-freeze the yeast pellet. The frozen pellet can be kept at −80 °C for future use.

### Step 2: protein purification using calmodulin-agarose beads

#### Material

• Calmodulin-agarose resin (Sigma- Aldrich, Cat# P4385).
• Dry ice pellets.
• Coffee grinder (or Pestle and mortar).
• Clean 15 mL and microcentrifuge tubes.
• Calmodulin binding *10X stock buffer* (2 M Tris-HCl (pH 7.9), 5 M NaCl, 1 M CaCl_2_, 10% Triton-X100).
• Calmodulin wash *10X stock buffer* (2 M Tris (pH 7.9), 5 M NaCl, 1 M CaCl_2_).
• Calmodulin elution buffer *10X stock buffer* (2 M Tris-HCl (pH 7.9), 1 M NaHCO_3_, 0.2 M EGTA, 7 μL β-mercaptoethanol in 10 mL buffer).
• Protease inhibitor cocktail.

Note: All purifications are performed at 4 °C with cold buffers and equipment also pre-chilled to 4 °C.

#### Procedure

1 Using a hammer, carefully break the frozen flacon tube to reach the pellet. Transfer the latter in a coffee grinder together with a similar size dry ice pellet.
2 Perform lysis by grinding the yeast cell pellet for approximately 30 s.
3 Transfer the lysed cell powder to ultracentrifuge tube and re-suspend in 10 mL cold calmodulin binding buffer (4 °C) with 25 μL CaCl_2_ and protease inhibitor cocktail.
4 Centrifuge cell lysate at maximum speed for 1 h at 4 °C. The supernatant is used for the purification step.

Alternate lysis procedure:

For smaller samples, the yeast cell pellets can also be lysed with glass beads using the following protocol:

• Re-suspend the frozen yeast cell pellet in about 200 μL lysis buffer (50 mM Tris−HCl (pH 8.0), 1% DMSO, 100 mM NaCl, 1 mM EDTA, 1 mM PMSF, 1 μg/mL leupeptin, 1 μg/mL pepstatin A).
• Add 1–3 g of pre-washed glass beads per gram of cell pellet
• Vortex the cells with glass beads 3–5 times for 1 min intervals. Keep the cells on ice.
• Centrifuge cells at 5000 rpm for 10 min. at 4 °C.
• Carefully transfer the supernatant to a new tube and carry on to purification step.
• Add about 200 μL of calmodulin beads to a 15 mL culture tube and wash beads twice with 1X calmodulin binding buffer. Carefully discard the supernatant using a pipette, taking care not to lose the beads.
• Transfer the supernatant from the previous step to the beads and incubate at 4 °C with gentle rocking for 3 h to overnight (optimum 5 h r).
• After the incubation period, centrifuge tube at 3000 rpm and 4 °C for 5 min.
• Using a 1 mL pipette, carefully discard the supernatant.

Note: Cut the end of the pipette tip to avoid breaking calmodulin resin.

9 Wash the beads twice with pre-chilled (4 °C) 300 μL 1X calmodulin binding buffer containing protease inhibitors followed by 2x washes with 300 μL 1X calmodulin wash buffer.
10 The proteins are eluted from the resin in 3 fractions of 200 μL 1X calmodulin elution buffer into fresh 1.5 mL microcentrifuge tubes.
11 The eluates are run on SDS-PAGE gel.

### Step 3: regeneration of calmodulin-agarose beads

CBP binding to the calmodulin resin is calcium-dependent. In the presence of Ca^2+^ ions, calmodulin undergoes a conformational change as four ions binds to their designated regions in the protein [13]. This results in an increased flexibility and the exposure of hydrophobic regions in the globular protein, allowing interaction between calmodulin resin and the CBP component of TAP-tag [13]. In the elution step, EGTA is used to chelate Ca^2+^, forcing calmodulin to regain its original conformation, thereby releasing the bait protein. The protocol described below includes washes to ensure complete depletion of Ca^2+^ ions to remove any remaining bound contaminating proteins as well as an equilibration step to restore the structural and functional properties of the calmodulin-agarose resin.

#### Materials

• Snake skin dialysis tubing (3.5 K MWCO, Thermo Scientific).
• M carbonate buffer with 2 mM EGTA (pH 7.9).
• M NaCl with 2 mM CaCl_2_.
• M acetate buffer with 2 mM CaCl_2_ (pH 4.4).
• Binding buffer (0.2 M Tris-HCl (pH 7.9), 0.5 M NaCl, 0.1 M CaCl_2_, 1% Triton-X100).

#### Procedure

**Fig. 2:**
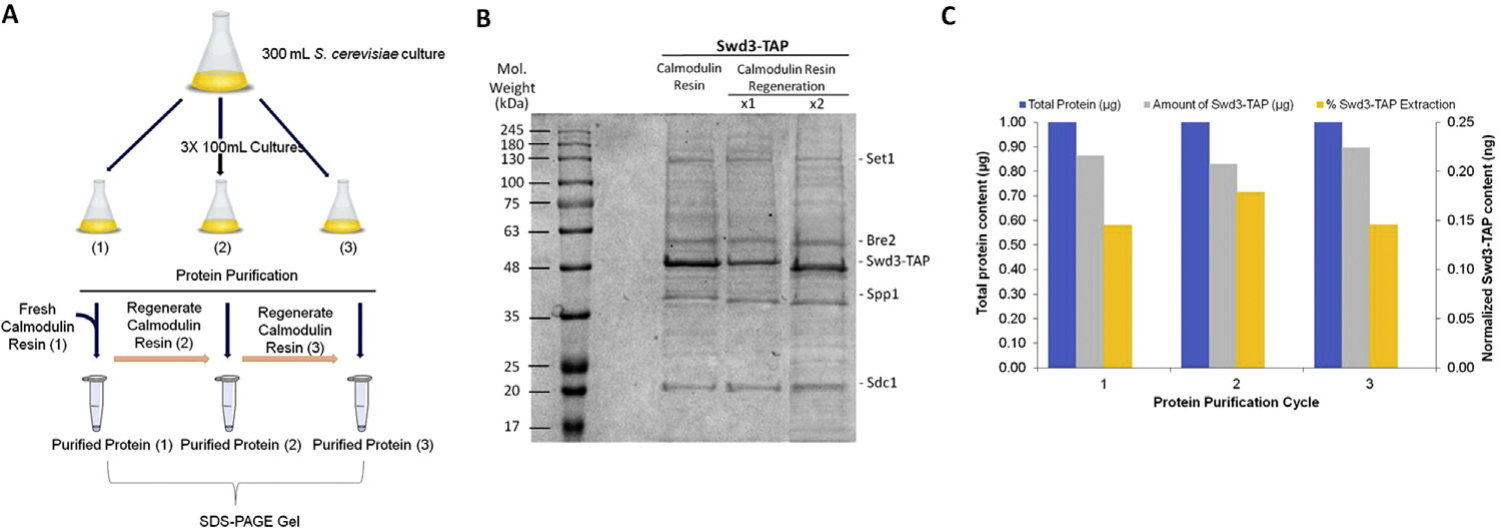
Protein purification of Swd3-TAP of the COMPASS complex using regenerated calmodulin beads. (A) Schematic flowchart describing the protocol that was used to track the efficiency of the calmodulin matrix. (B) Coomassie blue stained 10% SDS polyacrylamide gel used to visualize the purification of Swd3-TAP protein; associated members of the Swd3-associated COMPASS complex in *S. cerevisiae* are also successfully co-purified using single-step calmodulin-agarose resin. Purification of TAP-tagged Swd3 pulled-down other subunits of COMPASS including Set1 (124 kDa), Swd3-TAP (35 + 19.5 kDa), Spp1 (42 kDa), Bre2 (58 kDa) and Sdc1 (19.5 kDa). (C) Analysis of the total protein content in different fractions using BCA assay. The amount of protein was normalized to the total protein content in each purification cycle and the amount of Swd3-TAP protein and the percent of TAP-tagged protein extracted were plotted on a secondary axis.
2 Wash beads 3x with 300 μL 1 M NaCl with 2 mM CaCl_2._
3 Wash beads 3x with 300 μL 0.1 M acetate buffer (pH 4.4) containing 2 mM CaCl_2._
4 Equilibrate beads with 200 μL binding buffer for 2 h.
5 For long term storage, the beads are stored at 4 °C in 20% (v/v) ethanol.

1 Following protein purification, transfer the used calmodulin-agarose beads into a clean 1.5 mL microcentrifuge tube. Beads may be re-used at least 3 time without notable loss of binding (Fig. 2A). Dialyze 3 h to overnight using snake skin dialysis tubing in 0.1 M carbonate buffer (pH 7.9) with 2 mM EGTA.

#### Method validation

The method described above was effectively used to isolate Set1, the budding yeast ortholog of MLL1; a lysine methyltransferase (KMT) known to be over-expressed in leukemia and other cancers [14,15]. Set1 protein functions in the COMPASS complex which comprises of several other subunits required for integrity, stability and functionality of the protein [16]. In the present experiment, Swd3-TAP subunit was utilized for the isolation of the COMPASS complex from *Saccharomyces cerevisiae* at near physiological conditions. Fig. 2 presents the data obtained from the above described protocol. Fig. 2A shows the schematic diagram used to determine the efficacy of the calmodulin resin after 3 independent sets of purification and two subsequent regeneration cycles. Swd3-TAP together with some other subunits of COMPASS namely Set1, Spp1, Swd2 and Sdc1 were successfully extracted using single-step purification with calmodulin resin (Fig. 2B). Regenerating the calmodulin resin did not significantly affect its binding capacity nor its ability to pull-down interacting partners of the bait protein (Fig. 2B and C). The percent of Swd3-TAP protein extracted in each cycle is comparable after 3 rounds of purification.
